# Autonomic Nervous System Responses to Viewing Green and Built Settings: Differentiating Between Sympathetic and Parasympathetic Activity

**DOI:** 10.3390/ijerph121215026

**Published:** 2015-12-14

**Authors:** Magdalena M.H.E. van den Berg, Jolanda Maas, Rianne Muller, Anoek Braun, Wendy Kaandorp, René van Lien, Mireille N.M. van Poppel, Willem van Mechelen, Agnes E. van den Berg

**Affiliations:** 1 Department of Public and Occupational Health, EMGO Institute for Health and Care Research, Vrije Universiteit (VU) University Medical Center, van der Boechorststraat 7, NL 1081 BT Amsterdam, The Netherlands; riannemuller@hotmail.com (R.M.); anoek_braun@hotmail.com (A.B.); wendykaandorp@hotmail.com (W.K.); mnm.vanpoppel@vumc.nl (M.N.M.P.); w.vanmechelen@vumc.nl (W.M.); 2 Department of Psychology and Pedagogy, Vrije Universiteit (VU), Transitorium Van der Boechorststraat 1, NL 1081 BT Amsterdam, The Netherlands; jolanda.maas@vu.nl; 3 Department of Biological Psychology, Vrije Universiteit (VU), Transitorium Van der Boechorststraat 1, NL 1081 BT Amsterdam, The Netherlands; renevanlien@gmail.com; 4 Department of Cultural Geography, Faculty of Spatial Sciences, University of Groningen, Landleven 1, 9747 AD Groningen, The Netherlands; a.e.van.den.berg@rug.nl

**Keywords:** stress buffering, stress recovery, natural environment, autonomic nervous system, respiratory sinus arrhythmia, pre-ejection period

## Abstract

This laboratory study explored buffering and recovery effects of viewing urban green and built spaces on autonomic nervous system activity. Forty-six students viewed photos of green and built spaces immediately following, and preceding acute stress induction. Simultaneously recorded electrocardiogram and impedance cardiogram signal was used to derive respiratory sinus arrhythmia (RSA) and pre-ejection period (PEP), indicators of respectively parasympathetic and sympathetic activity. The findings provide support for greater recovery after viewing green scenes, as marked by a stronger increase in RSA as a marker of parasympathetic activity. There were no indications for greater recovery after viewing green scenes in PEP as a marker of sympathetic activity, and there were also no indications of greater buffering effects of green space in neither RSA nor PEP. Overall, our findings are consistent with a predominant role of the parasympathetic nervous system in restorative effects of viewing green space.

## 1. Introduction

Urbanization, as it is occurring all around the world, has been associated with an increase in stress-related diseases and mental disorders in people living in urban environments [1,2,3]. These developments increase the need for outdoor open spaces where urban residents can find relief from the demands of urban life and urban stressors such as noise and fear of crime and crowding [4,5]. As a result of urban expansion and densification such open spaces are becoming more and more scarce and no longer provide readily available, everyday resources for restoration from stress in urban environments. 

The concept of restoration refers to the process of a “return to unaffected affective, cognitive and psychophysiological functioning” [6]. There is increasing scientific evidence that particularly open spaces with natural or vegetated elements, e.g., green spaces, provide opportunities for restoration [7]. Numerous laboratory and field studies have shown that contact with real or simulated green settings as opposed to built settings has positive effects on mood, self-esteem and self-reported feelings of stress and depression, and can help to recover from stress and attention fatigue [8,9,10,11,12].

While most of the experimental work in this area has used affective and cognitive measures of restoration (e.g., [13]), several studies have demonstrated faster and more complete physiological restoration during exposure to green, as opposed to built, space, as indicated by cardiovascular and other physiological stress markers [9,14,15,16,17]. Importantly, exposure to real or simulated green space may not only promote recovery from stress, it may also increase stress resilience by attenuating or buffering the physiological response to a (future) stressor [18,19].

Physiological stress responses are regulated by the Autonomic Nervous System (ANS), a part of the nervous system not usually under voluntary control [20]. The ANS can be divided in a sympathetic and a parasympathetic branch. The sympathetic branch is linked with reactivity to the environment that regulates the fight-flight response, as indicated, among other things, by an increase in heart rate, myocardial contractility, and sweat production. The parasympathetic branch is linked with activity that causes slowing of the heart, increased heart rate variability, stimulation of salivary glands, and other responses that induce relaxation and help to compensate or buffer for periods of high stress. High resting levels of parasympathetic activity, or vagal tone, have been associated with numerous benefits including more adaptive emotion regulation strategies [21,22] and decreased risk of cardiovascular disease [23]. Furthermore, there are indications that individuals who show decreased parasympathetic or vagal control at times of stress and increased vagal control at times of rest display more adaptive social and emotional functioning [24,25]. Based on these findings, it seems important to learn more about the differential roles of parasympathetic and sympathetic pathways in restorative effects of green space. 

There are indications that the parasympathetic part may be dominantly involved in the restorative effects of green space [26,27,28,29]. For example, an early study by Ulrich and colleagues showed strong and sustained post-stress heart rate deceleration during the initial minutes of audio-visual exposure to videotapes of forests and water environments, which is consistent with dominant vagal responding [28]. More recently, Gladwell and colleagues demonstrated that parasympathetic activity was higher during viewing green as compared to built scenes, while there were no differences in sympathetic activity between the two viewing conditions [30]. Another study by the same group found that viewing green scenes prior to a stressor increased parasympathetic activity in the recovery period as measured by vagus-mediated heart rate variability (RMSSD) [26]. Notably, in the latter study, there were no signs of altered sympathetic activity in the recovery period. However, other studies have reported reduced sympathetic activity during or after exposure to green space, as indicated by, for example, decreased blood pressure, skin conductance, salivary cortisol and muscle tension [15,16]. One study showed that listening to sounds from nature after a stressful mental arithmetic task, as compared to listening to traffic and ambient noises, promoted a reduction of skin conductance level as a measure of sympathetic activation, but did not lead to greater parasympathetic activation as measured by heart rate variability [31]. 

In sum, although several studies suggest a dominant parasympathetic or vagal influence on stress recovery and stress buffering effects of green space, the empirical evidence is limited and somewhat inconsistent. Additional research is needed to further clarify the role of both—parasympathetic and sympathetic—parts of the autonomic nervous system in the stress regulating functions of exposure to green space. 

### The Present Research 

The present study consisted of a laboratory experiment in which we explored physiological stress responses during brief visual exposure to photos of urban green spaces compared to built spaces prior to stress-inducement (buffering) and after stress-inducement (recovery). We combined the standard paradigm for assessing stress buffering effects with the standard paradigm for assessing stress recovery in a within-subjects design. This design is similar to the design used by Brown *et al.* [26], with the exception that we added an additional block of viewing green or built scenes in between the stressor and recovery phase to sustain and strengthen the impact of the manipulation into the recovery phase. Activity of the ANS was measured by sophisticated cardiovascular measures that tap directly into sympathetic and parasympathetic pathways. Sympathetic activity of the ANS was measured by cardiac pre-ejection period (PEP), which reflects the time between the left ventricle contracting and the aortic valve opening [32]. An increase in sympathetic activity is associated with a decrease in PEP. Parasympathetic activity or vagal control was measured by peak-valley respiratory sinus arrhythmia (RSA), with an increase in vagal control associated with an increase in RSA [33]. PEP and RSA allow a more detailed study of autonomic nervous system responses than the commonly used cardiovascular measures such as heart rate and blood pressure [34]. Both measures were recorded continuously during the entire experiment to obtain a detailed view of the ANS response to the various tasks. 

We tested the following hypotheses:

(i) compared to viewing built scenes, viewing green scenes prior to exposure to a stressor buffers against stress, indicated by a smaller stress response during the stressor (smaller decrease in PEP and/or RSA);
(ii) compared to viewing built scenes, viewing green scenes after exposure to a stressor supports recovery from stress, indicated by a larger post-stressor recovery response (larger increase in PEP and/or RSA).

In a more exploratory vein, we examined whether buffering or recovery effects of green space, if they would occur, are more pronounced in the parasympathetic part of the ANS (as indicated by changes in RSA) or in the sympathetic part of ANS (as indicated by changes in PEP). 

## 2. Method 

### 2.1. Participants

Following ethical approval from the Medical Ethics Committee of the VU University Medical Centre, 60 students were recruited at the VU University via announcements on posters and flyers. Prior to the experiment, participants who had signed up were screened for exclusion criteria (smoking, use of heavy medication, self-reported chronic disease, current pregnancy). Ten participants were excluded due to smoking (*n* = 2), use of heavy medication (*n* = 5), and self-reported chronic disease (*n* = 3). An additional four participants were excluded after completing the experiment because of: failed electrocardiogram (ECG) recording (*n* = 4), leaving a total sample of 46 participants (25 females, mean age = 21 years). All participants enrolled in the study on a voluntary basis and gave their informed consent before they participated in the study. As a reimbursement for their participation participants received a monetary reward of 10 euro or 60 study credits.

### 2.2. Stimuli

**Figure 1:**
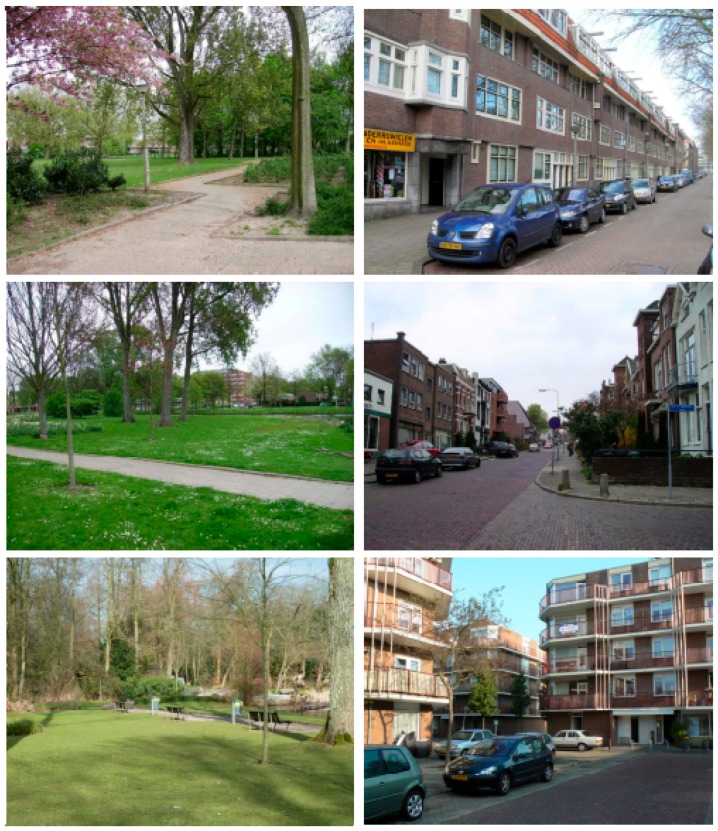
Examples of photos in the green and built stimulus sets.

Two sets of photos depicting green urban spaces, and two sets of photos depicting built urban spaces were created for the experiment (Figure 1). Each set consisted of 38 photos that were projected on a computer screen (28.8 cm × 51 cm) for 8 s each. Thus, the total viewing time for each set was about 5 min. Within each set the photos were always presented in the same order. The photos of green spaces primarily depicted urban settings with ample greenery such as parks, gardens, leisure areas and grassy fields, which tend to be rated high on restorativeness [35]. The photos of built spaces primarily depicted houses along roads in urban residential and inner-city areas with little greenery, which tend to be rated low on restorativeness [36]. In line with previous studies [37], none of the photos showed people or animals and they barely showed water. Furthermore, all four sets depicted a mixture of different seasons and different sunlight intensities. 

### 2.3. Stress Task

Psychological stress was induced using the Montreal Imaging Stress Test (MIST). This test exposes participants to challenging mental arithmetic problems presented on a computer screen, to which they had to respond by choosing a one-digit number from a rotary dial [38]. The MIST has been successfully used in previous studies to induce stress as measured by cardiovascular responses [39] and by changes in endocrine markers like dopamine and cortisol [38,40]. Each participant performed two versions of the MIST-task: an experimental (stress-inducing) version of the task and a control version. In the experimental version, the difficulty of the arithmetic problems was automatically adapted to the user performance to be just beyond the individual’s capacity, as measured in the control task. To further increase stress, a socio-evaluative threat component was built into the program. A mock performance indicator suggested poor performance on the participant in comparison to the average user, and an unpleasant sound with increasing pitch was played. In the control version of the MIST, the arithmetic problems were presented without time pressure and feedback components. 

### 2.4. Design

**Figure 2:**
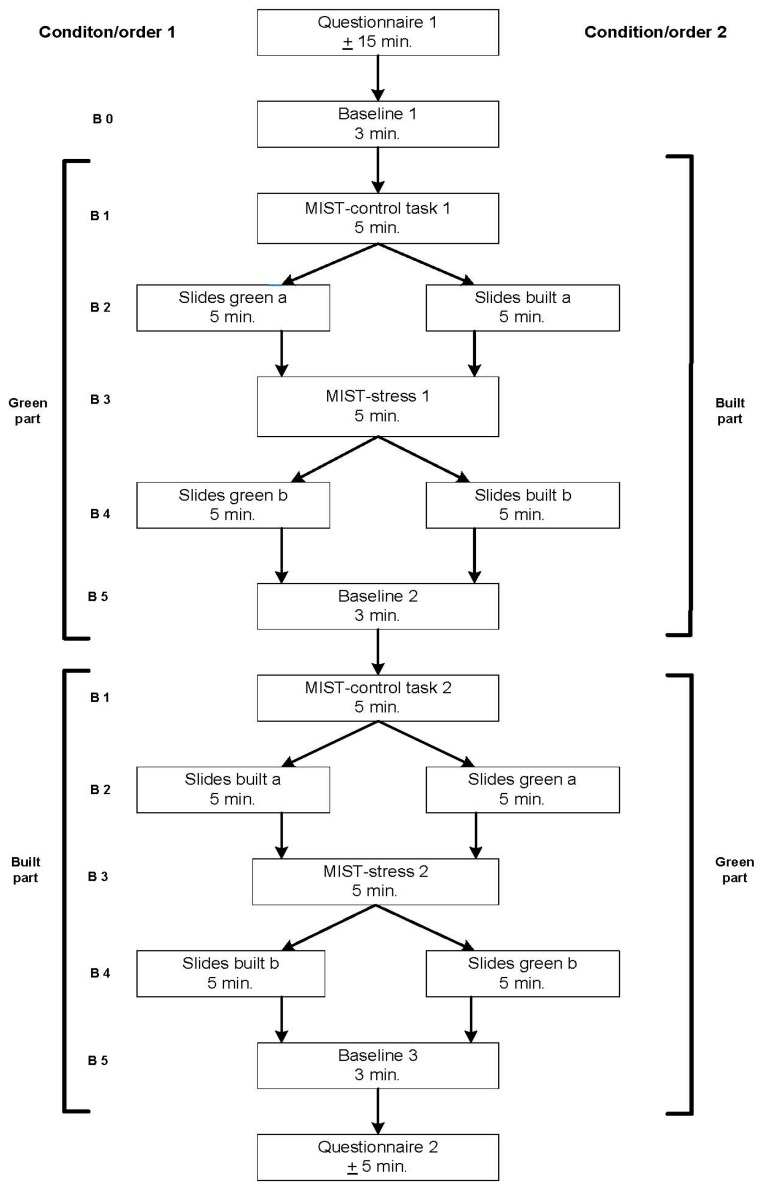
Design of the experiment.

A within-subject cross-over design was employed, where participants served as their own control. Figure 2 provides an overview of the experimental design with order (green first *vs.* built first) as a between subjects variable, and type of setting (green *vs.* built) and task (MIST stress *vs.* MIST control) as within subjects variables.

The experiment started with baseline measurements of RSA and PEP, followed by two parts, a “green part” and a “built part”. Within each part, the participant viewed two sets of green or built settings. Both the green and built part consisted of five blocks: (1) control version of MIST; (2) first time viewing of green or built scenes; (3) stressful version of MIST; (4) second time viewing of green or built scenes; and (5) follow-up baseline measurements. To minimize participant demands, the length of baseline measurements was kept to 3 min, following recommendations for measurements in resting position with the VU-AMS [41]. The length of the task blocks was set to 5 min because common practice research on restorative environments and stress suggests that this is an adequate minimum length for inducing restoration and stress. Participants were randomly assigned to the order of viewing green and built photos: 21 participants (11 females) started with the green spaces followed by the built spaces (condition 1); the other 25 participants (14 females) started with the built spaces followed by the green spaces (condition 2).

### 2.5. Measures

#### 2.5.1. Autonomic Response Measures: RSA and PEP 

The VU University Amsterdam Ambulatory Monitoring system (VU-AMS version 3.5, [41] was used to simultaneously record an ECG and ICG. These combined signals were used to extract the peak-valley respiratory sinus arrhythmia (RSA) and the pre-ejection period (PEP) that provide valid and preferred indicators of cardiac parasympathetic and sympathetic activity, respectively [42]. 

RSA measures the variation in heart rate that accompanies breathing. RSA is formally defined as the difference between the shortest inter-beat-interval (IBI) during inspiration and the longest IBI during expiration within a single breath [43,44]. Increases in RSA are thought to function as a “brake” on sympathetic influence on the heart, allowing the individual to rapidly regulate responses to environmental demands [45].

PEP is defined as the interval from the onset of left ventricular depolarization, reflected by the Q-wave onset (Q-onset) in the ECG to the opening of the aortic valve, reflected by the B-point in the ICG signal [46]. It has been determined that the length of this time period is almost entirely determined by sympathetic activation, and therefore, PEP can be described as an indicator of sympathetic activation of the heart. 

Faulty detected R-wave peaks were manually corrected or flagged as an artifact to ensure valid inter-beat interval (IBI) series. Mean RSA and PEP values were calculated for each of the eleven blocks of the experiment. Two researchers manually scored landmarks in the large-scale ensemble averaged electrocardiogram and impedance cardiogram (Q, B, C and X point) to extract the PEP-values [46,47]. The inter-rater reliability was 0.95, indicating a high agreement between the two researchers.

#### 2.5.2. Restoration Outcome Scale

As an indication of the perceived restorativeness of the scenes, each participant rated one built and one green setting on the Restoration Outcome Scale [48,49]. This validated scale consists of seven items that assess different dimensions of the restorative environment experience, including relaxation, attention restoration, clearing one’s thoughts, subjective vitality, and self-confidence. Sample items are “I feel calmer after being here” and “I can forget everyday worries here”. Each item was rated on a 5-point Likert scale ranging from 1 = not at all to 5 = completely. Reliability of the scale was sufficient for both the built scenes, Cronbach’s alpha = 0.79 and the green scenes, Cronbach’s alpha = 0.83. For each participant, mean scores on the ROS were calculated for each type of scene as the average of the seven items. 

### 2.6. Procedure

Upon arrival in the lab, participants were asked to read and sign an informed consent form. They also filled in a questionnaire with background questions about possible covariates that are known to influence ANS activity, including current mood, health, chronic stress levels, exercise in the past 24 h, and socio-demographic characteristics. Thereafter, electrodes were attached to participants’ bodies and connected to the monitoring device that recorded the electrocardiogram and impedance cardiogram (ECG/ICG) simultaneously. After obtaining optimal signal quality, the participant was individually seated in a small, soundproof and neutrally painted single room with a computer screen and a keyboard and were given information and instructions about the experiment. Participants were asked to sit as quiet as possible and always in an upright position to avoid interference with the ECG/ICG signal, as changes in posture and movement influence ANS responses [50]. Furthermore, the experimenter mentioned that the participant would be monitored via a camera to verify that he or she was indeed sitting still and not moving in the chair. 

At the start of the experiment, participants were asked to imagine themselves in the environments shown on the photos. The experiment started with a baseline measure of three minutes viewing a blank slide with a fixation cross in its center. The experiment ended with a post-experiment baseline measure of three minutes. After the experiment the participants were shown one photo of the green spaces and one of the built spaces and asked to evaluate these photos on the Restoration Outcome Scale. The post-experimental questionnaire also contained some questions about participant’s experiences with nature and green space, which are not reported in this paper. Finally, the participant was thanked, paid and debriefed. The total duration of the experiment, including attachment of electrodes and signal testing, lasted about one hour. 

### 2.7. Analysis

Analyses were performed using SPSS version 20 (IBM, Armonk, NY, USA). Before testing the main hypotheses concerning buffering and recovery, a one-way analysis of variance (ANOVA) was used to test for baseline differences in RSA and PEP between the two conditions. All other analyses were conducted using repeated measures ANOVA. Differences in perceived restoration outcomes between the green and built scene were tested using a repeated measures ANOVA, with scene type as a within-subjects factor. The effect of presentation order on RSA and PEP was tested by a repeated measures ANOVA, with scene type (green, built) and block (1–5) as within-subject factors and presentation order (green-natural, natural-green) as a between-subjects factor. Because there were significant effects of presentation order, analyses of stress buffering and recovery effects were restricted to the first six blocks of the experiment, with scene type as a between-subjects factor, and baseline scores in Block 0 as a covariate. Covariate analyses indicated that age and intensive exercise in the last 24 h were negatively related to RSA responses. Therefore, these variables, if significant, were included as additional covariates in analyses of RSA. Stress buffering effects were tested by examining the impact of pre-stress viewing of green or built scenes (as a between-subjects factor) on within-subject changes in RSA and PEP from MIST control to MIST stress. Recovery effects were tested by examining the impact of post-stress viewing of green or built scenes on changes in RSA and PEP from MIST stress to baseline 2. Finally, in a more exploratory manner, differences between the green and built groups in changes in RSA and PEP from baseline 1 to baseline 2 were examined as an indication of the overall impact of participating in the experiment on autonomous nervous system responses. 

## 3. Results

### 3.1. Perceived Restoration Outcomes

A one-way ANOVA revealed that the green scene was perceived as more restorative, M = 3.13, SD = 0.69, than the built scene, M = 1.64, SD = 0.5, *F*(1,45) = 153.61, *p* < 0.001, *η_p_*^2^ = 0.77. This finding is consistent with our *a priori* classification of the green scenes as more restorative than the built scenes. 

### 3.2. RSA

During the first baseline measurement, RSA was somewhat higher in the group who was assigned to viewing built scenes first than in the group who was assigned to viewing green scenes first. However, this difference was not significant, *p >* 0.35, *η_p_*^2^ = 0.02. A repeated measures ANOVA with Huynh-Feldt corrected df and baseline measures as a covariate revealed a significant interaction effect between presentation order (green-built, built-green), scene type (green, built), and block (B1–B5) on RSA, *F*(3.9,167.88) = 2.45, *p* = 0.05, *η_p_*^2^ = 0.05 (See Table S1 and Figure S1 in the supplementary file). Participants who had first viewed built scenes showed smaller changes in RSA between the blocks in the second phase than in the first phase of the experiment. This decreased responsiveness to the tasks might be a carry-over effect from viewing the built scenes in the first phase, which may have been more exhausting than viewing green scenes. However, the decreased responsiveness could also reflect a direct impact of viewing the green scenes. In general, the presence of order effects implies that the RSA data from the second phase of the experiment cannot be reliably interpreted in terms of exposure to green or built scenes, and data analysis will be limited to the first six blocks of the experiment (from first to second baseline measurements). 

#### 3.2.1. Effects of Scene Type on RSA 

Across the two conditions, RSA was generally higher during the photo viewing blocks (Block 2 and Block 4), adjusted mean = 100.21 ± 3.8, than during the MIST control and stress tasks (Block 1 and Block 3), adjusted mean = 79.46 ± 3.34, *F*(1,43) = 5.94, *p* = 0.02, *η_p_*^2^ = 0.12. During blocks of photo viewing and the MIST tasks, there were no significant differences in RSA between the green and built conditions *p*-values > 0.17. These findings suggest that, during the first phase of the experiment, viewing photos was generally experienced as more relaxing than performing the MIST tasks, independent of the content of the photos.

Contrary to our expectations, participants who viewed green scenes prior to the stressful version of the MIST did not show a weaker decrease in RSA from MIST control in Block 1 to MIST stress in Block 3 than participants who viewed built scenes, *F*(1,43) = 0.61, *p* > 0.44, *η_p_*^2^ = 0.01, after adjustment for baseline scores. Adjusting for additional covariates such as age and recent exercise levels did not change these results. Thus, there was no support for a greater stress buffering effect of viewing green scenes compared to viewing built scenes.

**Figure 3:**
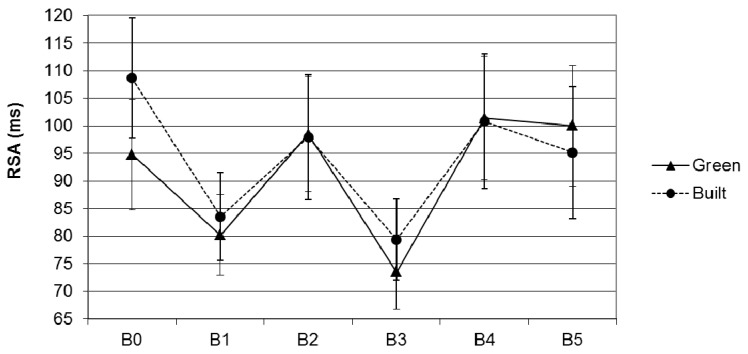
Unadjusted mean RSA during the first six blocks in the green and built condition. B0 = Baseline 1; B1 = MIST control; B2 = photo viewing 1; B3= MIST stress; B4 = photo viewing 2, B5 = baseline 2; error bars represent standard errors of the mean.

As shown in Figure 3, participants who viewed green scenes during recovery in Block 4 showed more increase in RSA from Block 3 to Block 5 than participants who viewed built scenes. After adjustment for baseline scores, this difference in recovery between the conditions, as indicated by the interaction between scene type (green, built) and block (3, 5) on RSA, was marginally significant, *p* = 0.08, *η_p_*^2^ = 0.1. When age and recent exercise levels were included as additional covariates, the difference in recovery between the green condition, mean adjusted change = 31.82 ± 7.02, and the built condition, mean adjusted change = 11.39 ± 6.41, was significant, *F*(1,41) = 4.45, *p* = 0.04, *η_p_*^2^ = 0.1. These findings are consistent with our hypothesis that viewing green scenes supports recovery from stress. 

In general, participants who viewed green scenes in the first phase of the experiment started with higher stress levels (as indicated by lower RSA values) than participants who viewed built scenes, while they ended with lower stress levels (higher RSA values) than participants in the built condition. Without adjustment for covariates, this difference was marginally significant, *p* = 0.1, *η_p_*^2^ = 0.06 After controlling for age and recent exercise, the difference in change in RSA from baseline 1 to baseline 2 in the first phase of the experiment between the green and the built condition was significant, *F*(1,42) = 6.18, *p* = 0.02, *η_p_*^2^ = 0.13. Thus, in general, the first half hour of the experiment was relaxing for participants exposed to the green scenes, mean adjusted change = 7.87 ± 6.88 while it was stressful for participants exposed to the built scenes, mean adjusted change = −15.67 ± 6.29. 

### 3.3. PEP

During the first baseline measurement, PEP was somewhat higher in the group who was assigned to viewing green scenes first than in the group who was assigned to viewing built scenes first. However, this difference was not significant, *p >* 0.47, *η_p_*^2^ = 0.01. A repeated measures ANOVA with baseline measures as a covariate revealed a significant effect between presentation order (green-built, built-green) and scene type (green, built, on PEP, *F*(4,172) = 3.07, *p* = 0.02, *η_p_*^2^ = 0.07 (See Table S2 and Figure S2 in the supplementary file). In the second phase of the experiment, participants in both conditions were generally less responsive to the MIST tasks and the green scenes than in the first phase. Because of this order effect, data analysis was restricted to the first six blocks of the experiment (from first to second baseline measurements). 

#### 3.3.1. Effects of Scene Type on PEP

Across the two conditions, PEP was somewhat higher during the photo viewing blocks (Block 2 and Block 4), adjusted mean = 111.83 ± 0.61, than during the MIST control and stress tasks (Block 1 and Block 3), adjusted mean = 107.87 ± 1.02, *F*(1,43) = 3.62, *p* = 0.06, *η_p_*^2^ = 0.08. During blocks of photo viewing and the MIST tasks, there were no significant differences in PEP between the green and built conditions *p*-values > 0.69. Thus, the PEP data also indicate lower stress levels during photo viewing than during the MIST tasks, independent of the content of the photos.

**Figure 4:**
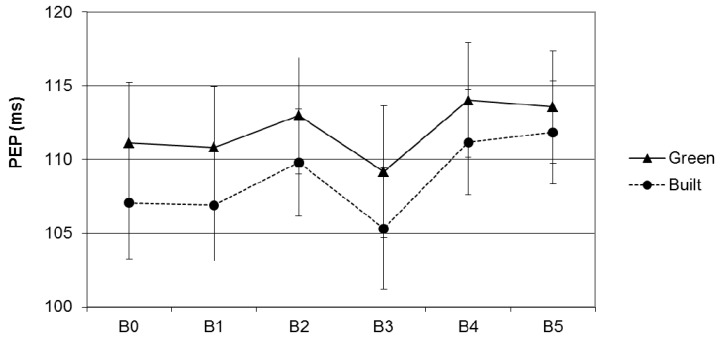
Unadjusted mean PEP during the first six blocks in the green and built condition. B0 = Baseline 1; B1 = MIST control; B2 = photo viewing 1; B3= MIST stress; B4 = photo viewing 2; B5 = baseline 2; error bars represent standard errors of the mean.

Contrary to the expectation, the change from MIST control in Block 1 to MIST stress in Block 3 did not differ between green and built conditions, *F*(1,43) = 0.02, *p* = 0.9, *η_p_*^2^ = 0.0. Thus, the PEP data do not support a greater stress buffering effect of viewing green scenes compared to viewing built scenes. As shown in Figure 4, PEP generally increased from MIST stress in Block 3 to the second baseline measurements in Block 5 across both conditions, *F*(1,43) = 5.5, *p* = 0.03, *η_p_*^2^ = 0.11. Contrary to our expectations, participants who viewed green scenes after the stressful MIST task did not show greater recovery than participants who viewed built scenes, *F*(1,43) = 0.55, *p* = 0.46, *η_p_*^2^ = 0.01. There were also no differences between the conditions in the change in PEP from baseline 1 to baseline 2, *F*(1,44) = 2.25, *p* = 0.14, *η_p_*^2^ = 0.05. Adjustment for covariates did not change the estimates of the impacts of condition on PEP. Thus, the PEP data do not provide support for greater stress buffering or recovery effects of viewing green scenes as compared to built scenes.

## 4. Discussion

The aim of this study was to investigate stress buffering and recovery effects of viewing urban green and built spaces and the role of the parasympathetic and sympathetic nervous system in these effects. To this end, a cross-over experiment was conducted in which participants’ electrocardiogram and impedance cardiogram signals were recorded during a series of tasks that involved viewing green and built scenes prior to and after performing challenging mental arithmetic tasks. The findings provide support for greater recovery in participants who viewed green scenes in the first part of the experiment as compared to participants who viewed built scenes. This recovery was marked by a stronger increase in RSA as an indicator of parasympathetic activity or vagal control. There were no indications for greater recovery after viewing green scenes in PEP as an indicator of sympathetic activity, and there were also no indications of greater buffering effects of green space in either the RSA or the PEP data.

Overall, the findings of this study point to a predominant role of the parasympathetic nervous system in recovery from stress after exposure to green space. This is mostly in line with the results of previous studies using ANS measures [26,27,28,30]. In particular, our findings resonate with a recent study by Brown *et al.* [26], who also reported that parasympathetic activity was only greater after viewing nature scenes in a period of recovery, and not during viewing of green scenes, or during stress exposure following viewing green scenes. Viewing green scenes may thus be particularly effective in supporting relaxation and recovery after experiencing a stressful period, and thereby could serve as an opportunity for micro-restorative experiences and a promising tool in preventing chronic stress and stress-related diseases.

### 4.1. Strengths and Limitations

To the extent of our knowledge, this is one of the first laboratory studies that combined buffering and recovery paradigms to simultaneously explore parasympathetic and sympathetic nervous system responses to viewing urban green and built spaces. The main strengths of the study are the use of a well-validated stress task (Montreal Imaging Stress task) and the objective assessment of stress responses with state-of-the art biomarkers (RSA and PEP). The study used a cross-over, within-subjects design, in which participants acted as their own control (they viewed both green and built scenes, in different orders). Advantages of this design are that between-subject variability can be ruled out and that high statistical power is gained with fewer participants. However, in the current study the cross-over design turned out to be a weakness. Participants’ responses showed significant order effects, and thus the data from the second part of the experiment could not be reliably interpreted. Data analysis had to be restricted to the first, between-subjects, part of the experiment, which decreased the statistical power of the design. 

Overall, there was less between-task variation in RSA and PEP in the second part of the experiment than in the first part, indicating a decreased responsiveness to the tasks. There are several explanations for this decrease in responsiveness. It is possible that participants guessed the aim of the study and realized that the difficulty of the MIST-task was adjusted by the computer program regardless of their competence. As a consequence of this so-called “sensitization” they may have lost their motivation during the second part of the experiment. Alternatively, participants may have simply become fatigued and/or bored over the course of the experiment, which lasted about one hour in total. Thus, sensitization together with fatigue and boredom may have attenuated the ANS responses in the second part. Interestingly, participants who viewed green scenes in the first part of the experiment showed less diminishment of RSA responsiveness in the second part than participants who first viewed built scenes. This finding suggests that impacts of sensitization, fatigue and boredom may be moderated (or buffered) by exposure to green spaces.

In the current study, exposure to the green and built spaces was simulated by photos. Although the use of photos is common practice in restorative environments research, there has been some criticism on the selection of scenes in previous studies [36,51]. Some studies have compared “beautiful green” *vs.* “ugly built”, which resulted in a comparison of aesthetics rather than green *vs.* built environments. To minimize this potential bias, we took care that the contents of the photos were representative of daily living environments and were not aesthetically spectacular. However, as a side-effect of this standardization, the visual stimuli may have been too weak and uninteresting for students, perhaps even boring if viewed a second time. 

Finally, we did not follow general requirements that HRV measurements should be segmented in equal blocks of five minutes [52]. However, because RSA, unlike other measures of HRV like SDNN, is not time-dependent, this lack of standardization is not a major problem. We compared changes in RSA from one block to another block of different length between conditions of viewing natural and built settings. Therefore, in each of the two conditions, the change in RSA is measured in exactly the same way, and any difference between the conditions can be reliably interpreted. 

### 4.2. Suggestions for Future Research

Future experimental studies on impacts of viewing green and built space on ANS responses should take into consideration that these impacts may extend over longer periods. Therefore, the experimental design should allow sufficient time for post-stress measurement. It is also recommended that baseline and experimental measurements are of similar duration [52]. If a cross-over design is employed, the first and second phase of the experiment should preferably be carried out on different days, with ample time in between, to avoid carry-over effects. 

While the present study focused exclusively on visual contact with green and built settings, future studies may investigate the relative contributions of visual, olfactory and auditory stimulation on restorative effects of exposure to urban green spaces [53]. The use of virtual reality techniques could provide more realistic and interesting visual simulations than photos, which could help to prevent boredom and fatigue [54]. By systematically varying the design and management characteristics of the green spaces, the outcomes of this type of fundamental research will become more relevant for urban planning and therapeutic practice [55]. Finally, by combining cardiovascular measures with other psychophysiological measures, like cortisol or self-reported mood [26], a more comprehensive understanding of restorative environment experiences may be obtained. 

## 5. Conclusions

This study indicates that five minutes of viewing urban green space can support recovery from stress as shown in enhanced parasympathetic activity. These findings strengthen and deepen the growing evidence-base for health benefits of green space in the living environment [56]. In particular, the present findings point to the importance of visual access to green space in providing readily available micro-restorative opportunities [57]. However, more research with more viewing time scenarios, different types of green settings, and different groups of participants is needed to further elucidate our understanding of the physiological and psychological pathways leading from viewing green space to recovery from stress. 

## Supplementary Files

Supplementary File 1
