# Supplementary material for: Autonomic Nervous System Responses to Viewing Green and Built Settings: Differentiating Between Sympathetic and Parasympathetic Activity
Source: Int J Environ Res Public Health. 2015 Dec 14;12(12):15860–74. doi: 10.3390/ijerph121215026 (PMC4690962; doi:10.3390/ijerph121215026)
Supplement: Supplementary File 1 [file ijerph-12-15026-s001.pdf]

Autonomic Nervous System Responses to Viewing Green and Built Settings: Differentiating Between Sympathetic and Parasympathetic Activity

Table S1. Mean RSA (with SD) during the 11 blocks.

	B0	First Phase					Second Phase				
**Condition/order**	**B0**	**B1**	**B2**	**B3**	**B4**	**B5**	**B1**	**B2**	**B3**	**B4**	**B5**
**Green-Built *M***	94.8	80.2	98.5	73.5	101.4	100.1	87.4	94.9	75.4	99.0	99.3
SD	44.8	34.3	54.8	27.1	59.1	55.2	37.5	48.5	26.1	50.6	50.0
**Built-Green *M***	108.7	83.6	97.9	79.4	100.8	95.2	90.4	91.4	86.4	103.6	98.7
SD	54.3	38.5	49.6	38.3	53.4	54.3	39.3	45.3	38.3	50.0	46.4

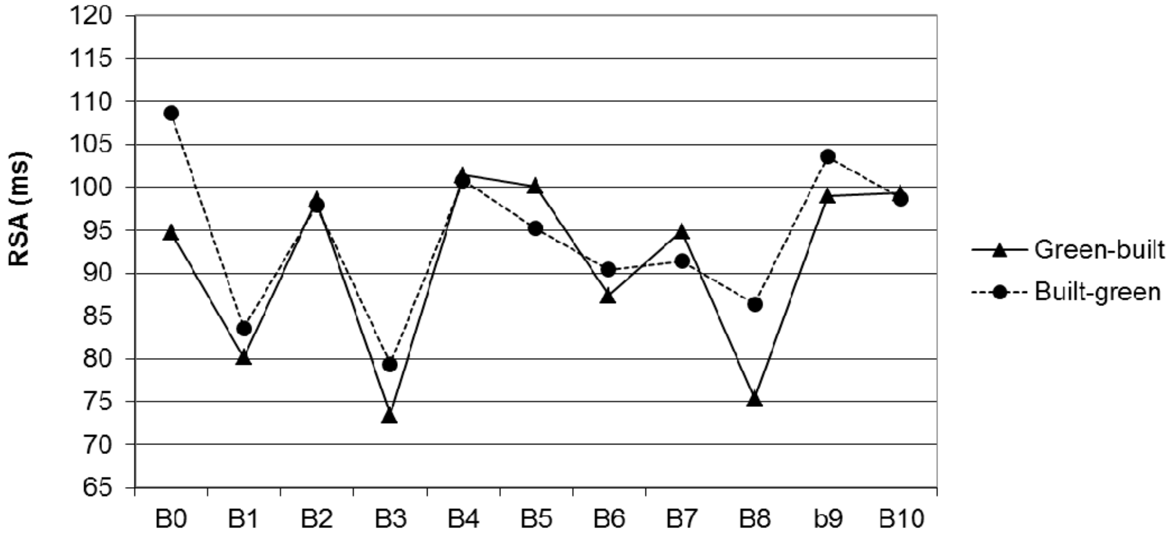

Figure S1. Mean RSA (msec.) in the 11 blocks of the experiment.

Table S2. Mean PEP (with SD) during the 11 blocks.

	B0	First Phase					Second Phase				
**Condition/Order**	**B0**	**B1**	**B2**	**B3**	**B4**	**B5**	**B1**	**B2**	**B3**	**B4**	**B5**
**Green-Built *M***	111.1	110.8	113.0	109.2	114.0	113.6	113.1	113.7	112.7	113.2	113.2
SD	20.8	21.8	19.1	22.2	20.3	19.3	20.0	20.0	20.1	17.8	16.9
**Built-Green *M***	107.1	106.9	109.8	105.3	111.2	111.8	110.8	111.5	111.5	112.9	113.0
SD	17.2	16.2	17.2	18.9	15.4	15.7	16.6	17.4	17.4	16.4	15.8

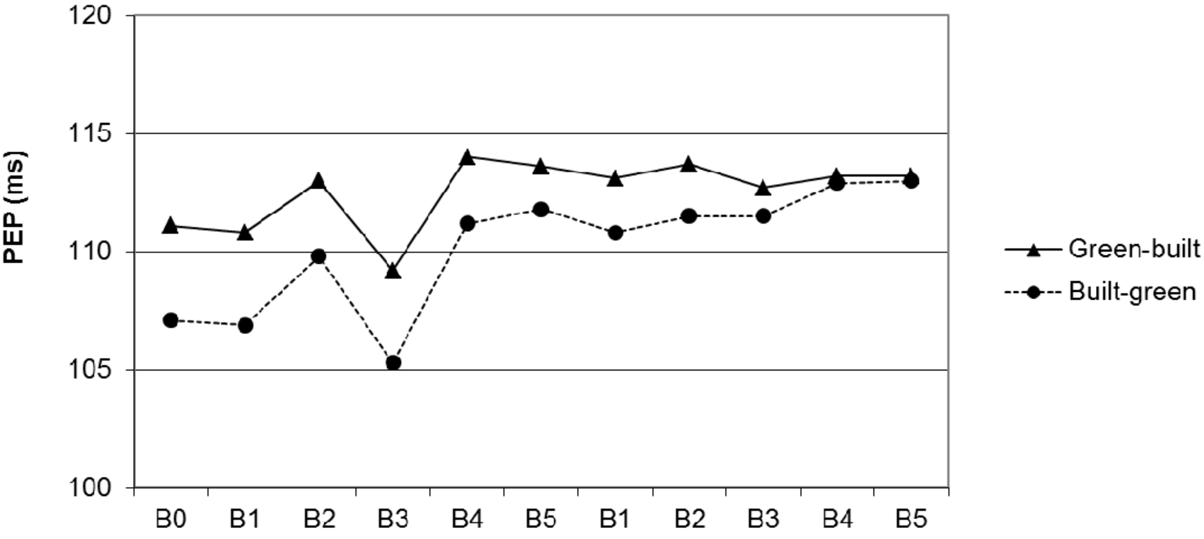

Figure S2. Mean PEP (msec.) in the 11 blocks of the experiment.

© 2015 by the authors; licensee MDPI, Basel, Switzerland. This article is an open access article distributed under the terms and conditions of the Creative Commons Attribution license (http://creativecommons.org/licenses/by/4.0/).
